# Inhibition of cathepsin B by caspase-3 inhibitors blocks programmed cell death in *Arabidopsis*

**DOI:** 10.1038/cdd.2016.34

**Published:** 2016-04-08

**Authors:** Y Ge, Y-M Cai, L Bonneau, V Rotari, A Danon, E A McKenzie, H McLellan, L Mach, P Gallois

**Affiliations:** 1 Faculty of Life Sciences, University of Manchester, Michael Smith Building, Oxford Road, Manchester M13 9PT, UK; 2 College of Marine Life Science, Ocean University of China, No. 5 Yushan Road, Qingdao, China; 3 Division of Plant Science, The James Hutton Institute, Invergowrie, Dundee DD2 5DA, UK; 4 Department of Applied Genetics and Cell Biology, University of Natural Resources and Life Sciences, Vienna, Austria

## Abstract

Programmed cell death (PCD) is used by plants for development and survival to biotic and abiotic stresses. The role of caspases in PCD is well established in animal cells. Over the past 15 years, the importance of caspase-3-like enzymatic activity for plant PCD completion has been widely documented despite the absence of caspase orthologues. In particular, caspase-3 inhibitors blocked nearly all plant PCD tested. Here, we affinity-purified a plant caspase-3-like activity using a biotin-labelled caspase-3 inhibitor and identified *Arabidopsis thaliana* cathepsin B3 (AtCathB3) by liquid chromatography with tandem mass spectrometry (LC-MS/MS). Consistent with this, recombinant AtCathB3 was found to have caspase-3-like activity and to be inhibited by caspase-3 inhibitors. AtCathepsin B triple-mutant lines showed reduced caspase-3-like enzymatic activity and reduced labelling with activity-based caspase-3 probes. Importantly, AtCathepsin B triple mutants showed a strong reduction in the PCD induced by ultraviolet (UV), oxidative stress (H_2_O_2_, methyl viologen) or endoplasmic reticulum stress. Our observations contribute to explain why caspase-3 inhibitors inhibit plant PCD and provide new tools to further plant PCD research. The fact that cathepsin B does regulate PCD in both animal and plant cells suggests that this protease may be part of an ancestral PCD pathway pre-existing the plant/animal divergence that needs further characterisation.

Programmed cell death (PCD) is relevant to many aspects of an organism's life and plants are no exception (reviewed in Drury and Gallois).^1^ The level of conservation of the PCD pathways across the different kingdoms of life is however not clear and plant PCD has specific cytological features that sets it apart from apoptosis.^2^ Despite the absence in plant genomes of orthologues for many key animal apoptosis genes including absence of caspase genes, proteases with caspase-like enzymatic activities were shown to be required for PCD by using synthetic caspase inhibitors (reviewed in Rotari *et al.*^3^ and Kacprzyk *et al.*).^4^ In particular, since its discovery 17 years ago,^5^ the importance of caspase-3-like enzymatic activity for plant PCD completion has been widely documented using caspase-3 inhibitors (reviewed in Bonneau *et al.*).^6^ Because of its prevalence, caspase-3-like activity is systematically used in the plant literature as a marker for PCD. However, the identity of the protease(s) responsible is only partially resolved. Metacaspases, the closest caspase homologues in plants, were clearly shown to be unable to cleave synthetic caspase substrates and to be insensitive to caspase inhibitors,^7^ despite controlling PCD. The *β*1 subunit of the 20S proteasome (PBA1) was shown in *Arabidopsis* to possess caspase 3-like enzymatic activity.^8^ This activity of PBA1 was confirmed in *Arabidopsis* by Gu *et al.*^9^ and in poplar by Han *et al.*^10^ Moreover, PBA1 was connected with PCD in *Arabidopsis* as downregulation of PBA1 blocked a fusion event between plasma and tonoplast membranes, an early stage of the hypersensitive response (HR) PCD induced by the pathogen *Pseudomonas syringae*.^8^ A proteasome requirement for PCD completion may however not be a general rule as other reports indicated that proteasome inhibition/downregulation could either promote PCD^11^ or reduce PCD,^12^ depending on the plant experimental system used. Because caspase-3 inhibitors had so far always been reported to repress PCD and never to induce it,^6^ it can be expected that additional pro-death proteases sensitive to caspase-3 inhibitors are present in plant cells.

In this work, we purified from *Arabidopsis* seedlings a protease with caspase-3-like activity that was identified using liquid chromatography with tandem mass spectrometry (LC-MS/MS) as *Arabidopsis thaliana* cathepsin B3 (AtCathB3). Recombinant and native AtCathB3 had enzymatic activity against the synthetic caspase-3 substrate DEVD (Asp-Glu-Val-Asp) and were inhibited by synthetic caspase-3 inhibitors. We propose here that caspase-3 inhibitors reduce PCD in plants by targeted cathepsin B since a *cathB*1-2-3 triple mutant has a reduced PCD after treatment using ultraviolet (UV), H_2_O_2_, methyl viologen (MV) and tunicamycin. These findings are reminiscent of the involvement of cathepsin B in mammalian PCD (reviewed in Rozman-Pungercar *et al.*^13^).

## Results

### Purification of a caspase-3-like activity from *Arabidopsis*

**Figure 1:**
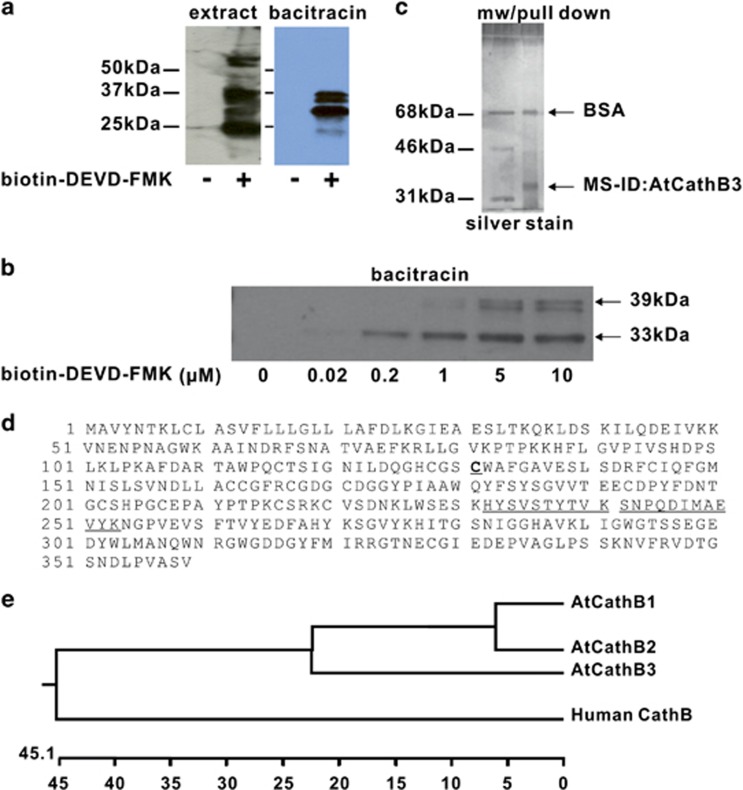
Identification of AtCathB3 in purified fraction containing caspase-3-like activity at pH 5. (**a**) ECL detection of proteins that interact with biotinylated caspase-3 inhibitor: biotin-DEVD–FMK in total *A*. *th*. leaf extract (extract); active fraction purified using bacitracin-sepharose affinity purification (bacitracin). kDa, molecular weight marker. (**b)** Bacitracin fraction labelled using increasing concentration of biotin-DEVD–FMK, from 0.02 to 10 *μ*M, and after incubation for 1 h at 37 °C the biotinylated proteins were detected by ECL. (**c**) Capture of the protease of interest. The purified DEVDase fractions were labelled with biotin-DEVD–FMK 1 *μ*M for 1 h at 37 °C and precipitated. After solubilisation, the biotinylated caspase-like protease was captured using streptavidin-agarose magnetic beads. After separation by electrophoresis, the proteins were visualised using silver staining and the major band was cut out and analysed. (**d**) AA sequence of AtCathB3; the two peptides identified by mass spec in the selected gel slice are underlined, and the catalytic cysteine C_131_ is in bold and underlined. (**e**) Phylogenetic tree of the CathB gene family in *A. th*. *AtcathB1*: At1g02300; *AtcathB2*: At1g02305; and *AtcathB3*: At4g01610 with human cathepsin B (P07858)

We have reported previously that UV-C radiation induced a caspase-3-like activity in *Arabidopsis* with an optimum around pH 5 and that UV-C-induced PCD could be totally blocked by the addition of the caspase-3 inhibitor Ac-DEVD-CHO.^14^ We therefore used that experimental system to identify the protease behind the caspase-3 activity detected using streptavidin pull-down after incubation of extracts with biotin-DEVD–fluoromethylketone (FMK). To reduce the complex profile of proteins identified in pull-downs directly from soluble protein extracts, we carried out first an affinity chromatography with bacitracin, a antibiotic cyclopeptide used successfully to purify plant cysteine proteases.^15, 16^ The bacitracin step introduced a 63-fold purification of the activity (Supplementary Table S1). In eluted and active fractions, biotin-DEVD–FMK labelled three major protein bands between 39 and 30 kDa that were already visible in labelled whole extract, although at a different intensity ratio (Figure 1a). A fourth labelled band at 25 kDa present in whole extracts did purify poorly (Figure 1a). The band *circa* 33 kDa was the most intense and labelled at a probe concentration as low as 0.2 *μ*M biotin-DEVD–FMK (Figure 1b). Subsequent pull-down of biotin-labelled proteins and silver staining revealed only a major protein at *circa* 33 kDa (Figure 1c). LC-MS/MS analysis of the band identified only two peptides corresponding to one protein: *Arabidopsis* cathepsin B3 (AtCathB3, At4g01610) (Figure 1d), one of the three *Arabidopsis* cathepsin B paralogues (Figure 1e): *AtCathB1* (At1g02300), *AtCathB2* (At1g02305) and *AtCathB3* (At4g01610).

### The caspase-3 inhibitor biotin-DEVD–FMK labels recombinant *Arabidopsis* cathepsin B

**Figure 2:**
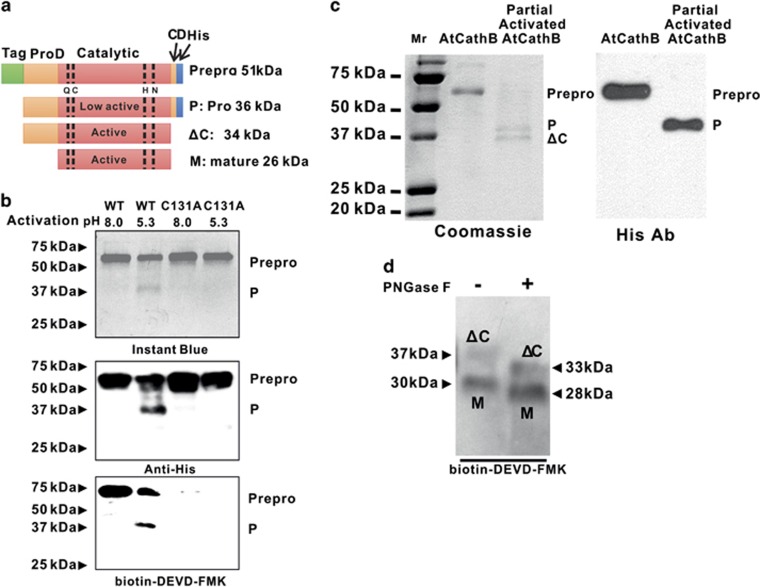
Caspase-3 inhibitor labels several processed forms of recombinant AtCathB3 *in vitro* and C_131_A abolishes labelling. (**a**) Cartoon of the recombinant AtCathB3 expressed in insect cells and its various processed forms: cherrytag (tag); prodomain (ProD); catalytic domain (catalytic); C-terminal prodomain (CD); his-tag in blue; the position the catalytic aa are indicated including C_131_ (Q,C,H,N). (**b**) Recombinant AtCathB3 wild-type and mutant C_131_A were affinity purified and partially activated at 70 *μ*g/ml, pH 8.0 or pH 5.3, at 4 °C. Aliquots were separated using SDS-PAGE and visualised by instant blue (top), incubated with anti-his antibody, ECL on western blot (middle) or activity labelling at pH 5 by 25 *μ*M biotin-DEVD–FMK and detected using streptavidin-HRP on western blot (bottom). (**c**) Recombinant AtCathB3 was partially activated at 200 *μ*g/ml, pH 5.5, at 8 °C, separated on a 15% SDS-PAGE and visualised using Coomassie blue (Coomassie) or transferred on Hybond P, incubated with anti-his antibody, followed by ECL detection. There were two processed forms: P and ΔC. N-terminal sequencing of P indicated a N-terminal at G27 of the native sequence (Supplementary Figure S2). (**d**) Full activation and de-glycosylation of AtCathB3: a fully processed form (M) was obtained at pH 4.5, 1 mg/ml, 10 *μ*g/ml dextran. Activated aliquots were labelled using 25 *μ*M biotin-DEVD–FMK (−). In addition, one labelled aliquot was deglycosylated using PNGase for 1 h at 37 °C (+). Labelled proteins were visualised by ECL detection on western blot using streptavidin-HRP.

Recombinant AtCathB3 was produced in insect cells both as a wild type (WT) form and as an inactive form with the catalytic cysteine mutated to alanine, C_131_A. Both forms were produced with an N terminal cherrytag (11 kDa heme-binding domain of cytochrome) and a C-terminal his-tag (Figure 2a and Supplementary Figure S1A) using a baculovirus vector that supports secretion of the recombinant protein into the culture media. Recombinant AtCathB3 was his-tag purified from the culture media before insect cell lysis by the virus vector. SDS-PAGE and Coomassie blue staining revealed a major band at 57–60 kDa corresponding to the AtCathB3 pre-proenzyme (Figure 2b) as confirmed using LC-MS/MS. AtCathB3 is predicted to have a N-pro-domain and a C-pro-domain that are processed during activation (Figure 2a). Obtaining full activation was found to be dependent on pre-proenzyme concentration, pH and the addition of dextran sulphate. To explain the various processed AtCathB3 forms labelled in whole plant extract and in purified fractions, we present here a succession of experiments to help identify processing intermediates in labelling profiles (Figure 2a). Incubation of the purified WT-recombinant prepro-AtCathB3 at 70 *μ*g/ml, pH 8, resulted in no processing; using pH 5 resulted in a processed band at ∼37–39 kDa (Figure 2b) and N-terminal sequencing showed that it corresponded to a pro-cathepsin form (P), with no N terminal cherrytag, starting in AtCathB position G_27_ (N terminus = GIEAES) and with the C-terminal his-tag still present (Figure 2b). Interestingly, both the full-length recombinant protein with the cherrytag and the partially processed pro-AtCathB3 were labelled with the caspase-3 inhibitor biotin-DEVD–FMK. This is consistent with the observation that the human pro-cathepsin B is partially active and can bind the E64-based probe DCG-04 (cysteine protease probe).^17^ In contrast, AtCathB3-C_131_A was inactive and did not label with biotin-DEVD–FMK (Figure 2b). Incubation at 200 *μ*g/ml, pH 5.5, yielded the pro-cathepsin form (P) and an additional intermediate form (ΔC), ∼3 kDa smaller, corresponding to C-domain-His-tag removal (Figure 2c). Finally, incubation at 1 mg/ml, pH 4.5, in the presence of 10 *μ*M dextran sulphate resulted in the ΔC form (37 kDa) and a mature form *circa* 30 kDa (M) that both reacted with biotin-DEVD–FMK (Figure 2d). The fact that the various forms migrate at a higher molecular weight (MW) than predicted from the sequence (Figure 2a) was in part due to *N*-glycosylation as de-glycosylation using the enzyme PNGase F resulted in an apparent molecular mass reduction of 4 kDa for the ΔC form and 2 kDa for the mature form (M) (Figure 2d). Glycosylation is expected to also occur in *Arabidopsis* as AtCathB3 has a predicted signal peptide for endoplasmic reticulum (ER) targeting.

### Recombinant *Arabidopsis* cathepsin B has caspase-3-like activity in enzymatic assays

**Figure 3:**
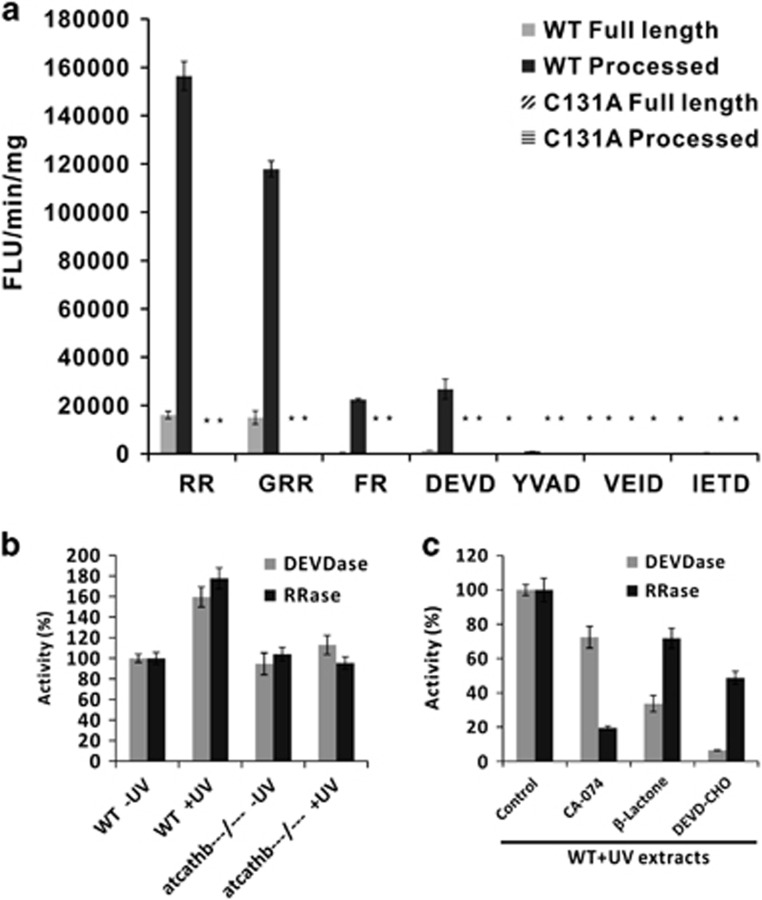
AtCathB3 has caspase-3-like enzymatic activity (DEVDase). (**a**) Substrate specificity of recombinant AtCathB3. Purified recombinant AtCathB3 wild-type and mutant C131A were activated and enzymatic assays carried out against cathepsin B substrates (RR, GRR, FR), caspase-3 substrate DEVD, caspase-1 substrate YVAD, caspase-6 VEID and caspase-8 IETD at 50 *μ*M final. Asterisk indicates zero activity detected. Activities are standardised against protein concentration. Error bars are S.D. of triplicate. (**b**) Enzymatic assays using DEVD and RR as substrates with protein extracts from WT Col-0 and *AtCathB* triple-mutant 7-day-old seedling untreated (−UV) or treated with 10 kJ/m^2^ UV-C treatment (+UV). Relative fluorescence units/min were measured at 24 h and calculated as a percentage of WT activity. (**c**) Relative contribution of cathepsin B and proteasome to caspase3-like activity (DEVDase) and RR activity in seedling extracts 24 h after 10 kJ/m^2^ UV-C treatment. Seedling extracts were pre-incubated in the presence of various protease inhibitors at the final concentration of 100 *μ*M (except 1 mM for CA-074) for 30 min at 30 °C. CA-074: cath B inhibitor; *β*-lactone: proteasome inhibitor; DEVD-CHO: caspase-3 inhibitor. The remaining activity was calculated as a percentage of the control (no inhibitors). Error bars indicate ±S.D. for triplicate

The optimal pH for recombinant AtCathB3 activity was found to be pH 5.5 (Supplementary Figure S1B). Enzymatic activity assays established that AtCathB3 had its highest activity against the human cathepsin synthetic substrate Arg-Arg (RR) and that papain substrates FR and GRR were cleaved less efficiently (Figure 3a). Crucially, AtCathB3 was capable of cleaving the caspase-3 substrate DEVD but had no activity against other caspase-like activities detected in plant extracts: caspase-6 substrate VEID (Val-Glu-Ile-Asp), caspase-8 substrate IETD (Ile-Glu-Thr-Asp) and very limited activity against caspase-1 substrate YVAD (Tyr-Val-Ala-Asp). The mutated AtCathB3-C_131_A was totally inactive against the substrates tested (Figure 3a) indicating that the activity detected was not due to contamination with proteases from insect cells. The three human cathepsin B inhibitors Ac-LVK-CHO, CA-074 and Z-FA-FMK and the caspase-3 inhibitor Z-DEVD-CHO were able to strongly inhibit the DEVDase activity of AtCathB3 (Table 1). The caspase-1/plant VPE inhibitor Ac-YVAD-CHO inhibited 60% of the activity.

### Cathepsin B contributes to the caspase-3-like activity detected in *Arabidopsis* extracts

To compare the recombinant enzymatic data with *in planta* data, we measured RRase and DEVDase activities in total protein extracts from WT and a triple-mutant line after a PCD-inducing UV-C dose.^14^ At 24 h after treatment, both RRase and DEVDase activities in WT increased by 50–70% of the untreated activity, whereas there was no significant induction in the triple mutant (Figure 3b). When extracts of untreated seedlings of the triple mutant, leaky for *AtCathB2*, were preincubated with the proteasome inhibitor *β*-lactone or the cathepsin B inhibitor CA-074 (Supplementary Figure S2), *β*-lactone inhibited 60% of the DEVDase activity and CA-074 20%. Both inhibitors combined left an activity of 20%. Pre-incubating UV-treated WT extract with CA-074 (Figure 3c) gave a DEVDase reduction of 30%, consistent with the induction data, whereas *β*-lactone reduced the activity by 70%, again consistent with the activation data. As expected, the inhibitor DEVD-CHO reduced DEVD by 95% (Figure 3c). In contrast, DEVD-CHO inhibited 50% of the RR activity, suggesting cathepsin B represented half of that activity (Figure 3c). This is consistent with the fact that the RR substrate can be cleaved by several proteases: cathepsin B, papains, metacaspases and probably others. This makes the inhibition of RRase activity by CA-074 and *β*-lactone difficult to interpret even more so as neither of these inhibitors have a narrow specificity. To conclude, in our conditions and at pH 5.5, the increase in caspase-3-like activity in *Arabidopsis* extracts after PCD induction appears primarily due to AtCathB, whereas the proteasome provided the larger part of the activity in noninduced conditions. In addition, DEVD-CHO appears to be an inhibitor of plant cathepsin B that is more specific than CA-074 as the latter inhibits RRase activity more than DEVD-CHO does.

### Biotin-DEVD–FMK labels several cathepsin B forms in *Arabidopsis* extracts

**Figure 4:**
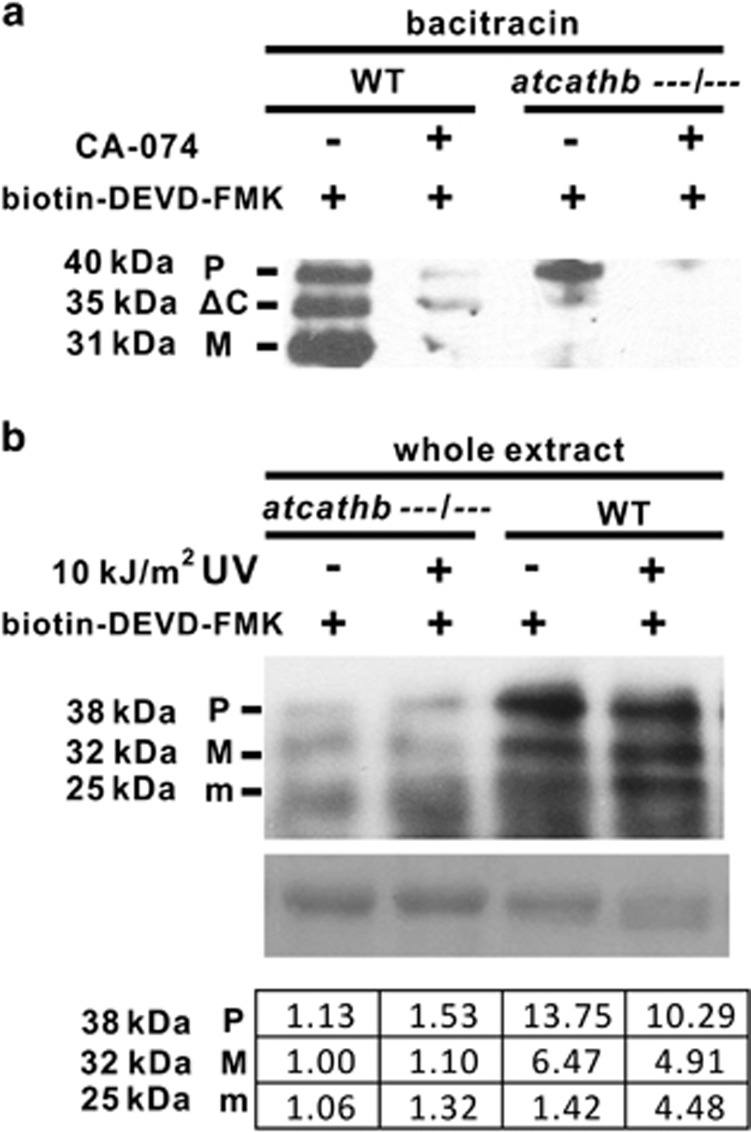
DEVD-FMK labelling *in planta* is abolished by cathepsin inhibitor CA-074 and *AtCathB* downregulation. Sizes in kDa are apparent sizes of the bands. Letters correspond to cathepsinB forms predicted from recombinant data: pro-enzyme (P); intermediate form with C-terminal domain processed (ΔC); mature form (M); short mature form (m). (**a**) Soluble proteins were extracted from 2-week-old *Arabidopsis* seedlings, WT and triple mutant (atcathb−−−/−−−) after UV-C irradiation and affinity purified using a bacitracin-sepharose column. DEVDase fractions were pre-incubated with or without 1 mM CA-074 at 30 °C for 30 min and then labelled with 10 *μ*M biotin-DEVD-FMK at 30 °C for 1 h. Equal volumes were loaded on SDS-PAGE gels and biotin labelling was detected using streptavidin and ECL. (**b**) The 4-day seedlings of Col-0 and triple mutant (atcathb−−−/−−−) were untreated (−) or treated with 10 kJ/m^2^ UV-C (+) to induce PCD. Soluble proteins were extracted 24 h after treatment, labelled using biotin-DEVD–FMK and separated using SDS-PAGE. Biotin labelling was detected using streptavidin-HRP and ECL. Rubisco large subunit (RbcL) stained on the corresponding membrane with ponceau S was used as a loading control (middle panel). The intensity of each band was measured using GelQuantNet (www.biochemlabsolutions.com). Data are presented as a ratio with the lowest intensity band set as 1.00

To match caspase-3-like activity labelling in plant extract with cathepsin B, we first pre-incubated bacitracin-purified DEVDase fractions with the cathepsin B inhibitor CA-074 before biotin-DEVD–FMK labelling. The three bands labelled in WT fractions were reduced dramatically by the inhibitor (Figure 4a), suggesting that all three labelled bands were due to AtCathB3 and/or its paralogues. This was confirmed genetically by labelling purified fractions from a cathepsin B triple-mutant line. The two lower bands were faint in the cathepsin B triple mutant, and the top band was very much reduced. The top band was subsequently eliminated by incubating with CA-074 (Figure 4a). This top band can be explained by the fact that the triple-mutant line is leaky for *AtCathB2*.^18^ Taking into account the labelled band sizes observed in the recombinant experiments, we propose that the three bands in purified fractions correspond by order of size to (1) pro-AtCathB (P), (2) a processing intermediate (ΔC) and (3) the mature AtCathB (M). To link this AtCathB activity labelling with the increased in DEVDase activity in seedling extracts, we labelled whole protein extracts with biotin-DEVD–FMK using untreated and UV-treated seedlings (Figure 4b). In the whole extract of untreated WT seedlings, three labelled bands between 38 and 25 kDa were detected that were only faintly present in the triple-mutant extract, suggesting that the three bands were due to AtCathB (Figure 4b). Only the two top bands matched bands from bacitracin fractions (P and M) with bacitracin (ΔC) being absent. We observed that the 25-kDa band (m) present in whole extract failed to bind to bacitracin, possibly because of poor solubility in the buffers used (Figure 1a). The 25-kDa band (m) was identified after streptavidin pull-down of whole extracts, gel silver staining and LC-MS/MS analysis of a 25-kDa gel slice as combined AtCathB2 and AtCathB3 (Supplementary Figures S3 and S4). The 25 kDa m form is therefore a processed mature form of AtCathB (M). This processing might require a protease only present in whole extracts or high local concentration of CathB as it was never observed in bacitracin-purified fractions or activated recombinant fractions. We concluded that the three *Arabidopsis* proteins labelled by DEVD–FMK in whole extracts and faint in the leaky triple mutant are three processing forms of the AtCathB protease that are all reactive with the DEVD activity probe. In untreated WT extracts, the top band at 38 kDa corresponding to the pro-enzyme (P) is the 3- to 10-fold more intense than the second and third band. In WT treated with UV-C treatment, the proenzyme decreased by 3.5 density units, whereas the lowest band (m) increased by a similar amount (3.1), suggesting that an activation of the proenzyme into a mature, more enzymatically active form is the main cause of the increased AtCathB activity detected in seedling enzymatic assays.

### Cathepsin B is required for abiotic stress-induced PCD in *Arabidopsis*

**Figure 5:**
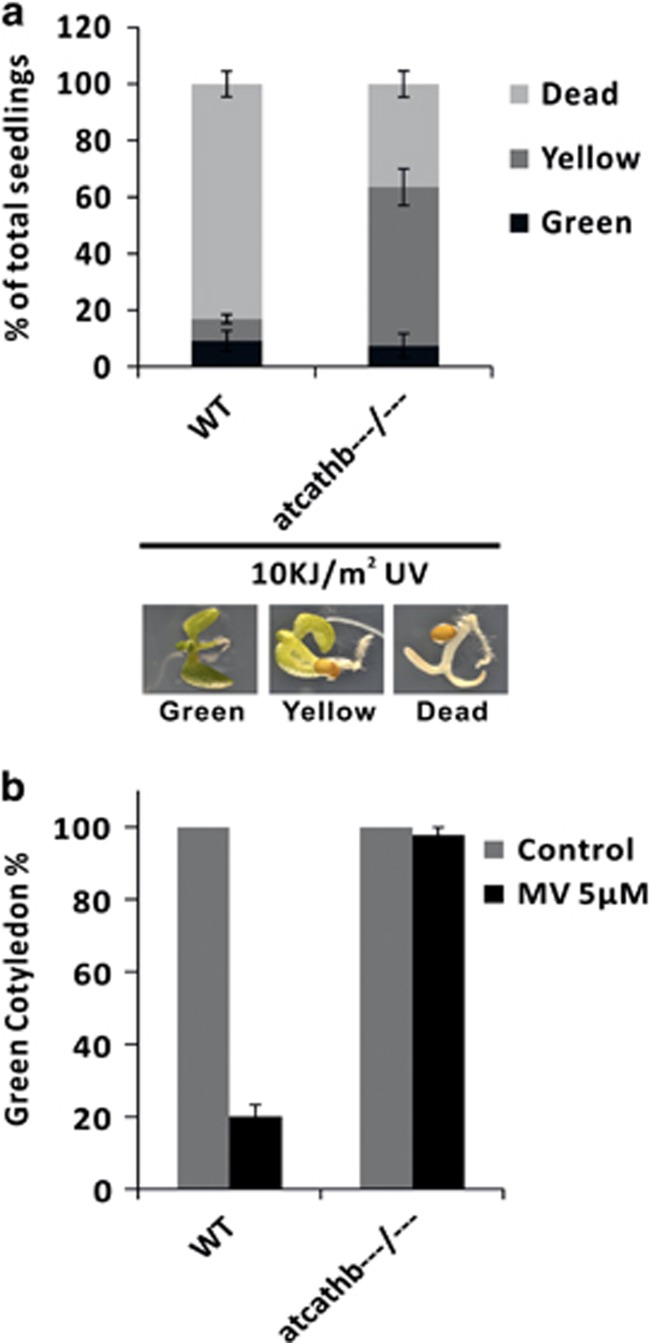
Reduced PCD phenotype of AtCathB KO lines submitted to abiotic stresses. (**a**) *Arabidopsis* seedlings from WT and cathepsin B triple-mutant line (atcathb−−−/−−−) were grown in MS medium for 4 days and then exposed to 10 kJ/m^2^ UV-C to induce PCD. Seedlings were observed 24 h after UV-C treatment and scored in three classes: green, yellow and dead as illustrated. Results are given as the percentage of seedlings of each category in the population (*n*=90). Error bars indicate ±S.D. for triplicate. (**b**) Sterilised seeds were germinated *in vitro* and grown in continuous light on MS medium (control) or MS supplemented with methyl viologen (MV) at 5 *μ*M. Seedlings with green cotyledons out of total seedlings were scored at 10 days. Error bars indicate ±S.D. for triplicate

Caspase-3 inhibitors have been shown to reduce plant PCD in many experimental systems. One explanation that comes out of our work is that caspase-3 inhibitors may inhibit plant PCD by inhibiting AtCathB activity. If cathepsin B mediates the effect of caspase-3 inhibitors on plant PCD, then genetic inactivation should phenocopy partially or totally the reduction of PCD observed after caspase-3 inhibitor application. We tested cathepsin B mutant lines in four PCD experimental systems where DEVDase activity had been reported. We first used a UV-C PCD assay using both 4-day-old seedlings and protoplasts.^14^ UV-C induced PCD in *Arabidopsis* with DNA laddering as well as fragmentation of the nucleus.^19^ This PCD was accompanied by reactive oxygen species (ROS) accumulation and loss of mitochondrial transmembrane potential^20^ and was totally inhibited by the application of a caspase-3 inhibitor to protoplasts.^14^ In WT, 10 kJ/m^2^ UV-C caused bleaching and PCD in 83% of seedlings (Figure 5a), whereas in the triple-mutant background, the PCD outcome was reduced down to 44%. When using protoplasts, 10 kJ/m^2^ UV-C induced PCD in 50% of WT protoplasts but no significant PCD in the triple-mutant protoplasts (atcathb−−−/−−−) (Supplementary Figure S5A). Single AtCathB KO lines for B1, B2 or B3 had no reduced PCD (data not shown), consistent with earlier reports that the three *Arabidopsis* CathB are redundant in HR.^18^ In a second experimental system, we used seedlings and the herbicide MV. MV acts inside chloroplasts, generating ROS that induce cell death with PCD hallmarks such as DNA laddering^21^ and cytochrome *c* release.^22^ PCD induction by MV is mediated by phytaspase in tobacco^22^ and metacaspase 8 in *Arabidopsis*.^23^ We germinated seeds on MS media supplemented with an optimised MV concentration of 5 *μ*M. At 10 days, only 20% of WT seedlings had cotyledons that remained green, whereas 98% of triple-mutant seedlings had green cotyledons (Figure 5b). Additional protoplasts assays were carried out (Supplementary Figure S5). We first tried H_2_O_2_-induced PCD as caspase-3-like activity was shown induced during the process.^24, 25^ In addition, Tiwari *et al.*^26^ showed that treating *Arabidopsis* cells with H_2_O_2_ induces a PCD characterised by cytochrome *c* release from mitochondria and inhibition by the cysteine protease inhibitor E-64.^27^ In Supplementary Figure S5B, no significant PCD was induced by 10 *μ*M H_2_O_2_ in protoplasts of the triple-mutant background as compared with nearly 100% PCD in WT. Finally, we assessed ER stress-induced PCD using tunicamycin as an inducer. ER stress-induced PCD is linked with H_2_O_2_ accumulation, cytochrome *c* release from mitochondria, chromatin condensation and a 2.5-fold induction of caspase-3-like activity in soybean suspension cells.^28^ In *Arabidopsis* roots, this PCD is linked with H_2_O_2_ accumulation, chromatin condensation and oligonucleosomal fragmentation of nuclear DNA.^29^ Finally, in *Arabidopsis* seedlings, tunicamycin induces a 10-fold increase in caspase-3-like activity.^30^ We found in the triple-mutant background that there was no significant PCD induced by tunicamycin as compared with 30% death in WT protoplasts (Supplementary Figure S5C). Taken together, the results indicate a major requirement for AtCathB in the PCD induced by abiotic stresses and support the suggestion that caspase inhibitors reduce PCD by inhibiting CathB. Besides these stress phenotypes, we did not observe any dramatic developmental defect in the triple-mutant plants, possibly implying that the presence of AtCathB is not a strict requirement for developmental PCD.

## Discussion

### Cathepsin B is a source of caspase-3-like activity in plants

Our study identified cathepsin B as a source of caspase-3-like activity in *Arabidopsis* extracts. This statement is supported by (1) LC-MS/MS identification of cathepsin B in partially purified fractions containing caspase-3 like activity; (2) caspase-3-like activity of the recombinant AtCathB3 produced in insect cells, (3) labelling inhibition of plant extracts with caspase-3 activity probes when applying a cathepsin B inhibitor, and (4) reduced caspase-3-like activity and caspase-3-like labelling in *AtCathB* triple-mutant extracts. We concluded that in plants, cathepsin B and the PBA1 subunit of the 20S proteasome are the two main contributors to caspase-3-like activity at *circa* pH 5. With the hindsight of this work, many studies measuring caspase-3-like activity in plants over the past 15 years have highlighted and established the implication of cathepsin B and the proteasome in plant PCD processes. CathB is not the major source of caspase-3-like activity in plant extract but nevertheless has a key role in PCD.

### Inhibition of PCD in plants using caspase-3-inhibitors targets cathepsin B

For a protease, to have a caspase-3-like activity in itself does not prove a regulatory role in PCD, only chemical or genetic ablation does. In the case of cathepsin B, we report a strong implication of *AtCathB1-2-3* in PCD induced by abiotic stress such as UV-C, oxidative stress (H_2_O_2_, MV) and ER stress, extending the role described for *AtCathB1-2-3* in PCD induced by biotic stresses such as in HR caused by *Pseudomonas* infection.^18^ Putting together the inhibition of AtCathB by caspase-3 inhibitors and the genetic requirement of *AtCathB* for PCD, we propose that when caspase-3 inhibitors downregulate PCD in plants, they can do so by inhibiting cathepsin B that in turn blocks the PCD pathway at a point yet to be determined. In other words, the interaction of cathepsin B with caspase-3 inhibitors explains the widely reported physiological effect of these inhibitors on plant PCD. UV-C-induced PCD would be the best example of this as the caspase-3 inhibitor treatments described in Danon *et al.*^14^ exactly phenocopied our *AtCathB* triple-mutant phenotype with a total block of PCD induction. As far as the proteasome is concerned, applying caspase-3 inhibitors will inhibit the subunit PBA1 and, as mentioned in the Introduction, this could either inhibit or induce PCD. Depending on the plant cell death pathway activated, the proteasome presumably degrades either a pro-PCD factor or an anti-PCD factor. When proteasome inhibition promotes PCD, AtCathB is possibly downstream of the proteasome and the main point of inhibition, as caspase-3 inhibitors consistently inhibit PCD. Further experiments will be required to investigate the possible relationship between the proteasome and cathepsin B and define the relative contribution of these enzymes to PCD execution in various experimental systems, including developmental PCD.

### A proposed model for the role of cathepsin B in plant PCD

One striking difference between plant cathepsin B and animal cathepsin B is that the plant protease has been shown *in vitro* to possess a higher endopeptidase activity than the human one.^31, 32^ This can be explained at the structural level by the fact that the plant sequence shows a much shorter so-called occluding loop region. This occluding loop affects substrate access to the catalytic site and plays a critical role in the exopeptidase activity of human cathepsin B.^33^ Because of this, it can be argued that plant cathepsin B is a potent endopeptidase rather than an exopeptidase. An efficient endopeptidase activity is compatible with the idea that plant cathepsin B may cleave target proteins during PCD in addition to having a role in protein degradation as suggested for example during seed germination.^34^

Studies with mouse cathepsin B KO lines gave robust evidence that CathB has a role in TNF-induced PCD of the liver.^35, 36^ Animal studies suggested that cathepsin B-mediated cell death proceeds mainly through the truncation of the protein Bid in the cytosol,^37^ resulting in the activation of the cell death mitochondria pathway (reviewed in Repnik *et al.*).^38^ Bid is a member of the Bcl2 family and there is no evidence that a homologue exists in plant genomes. However, the animal PCD model of a partial leakage of cathepsin B out of the lysosome into the cytosol, resulting in protein target activation or degradation,^39^ could be translated into a plant scenario where vacuole membrane permeabilisation would release cathepsin B into the cytosol where it may cleave/degrade a number of substrates, activating signalling towards PCD. This would be consistent with AtCathB having been reported as vacuolar in proteomic studies.^40^ An alternative and less favoured model would integrate the role of cathepsin B in the normal turnover of proteins, including a role during autophagy. This might be another route by which AtCathB inhibition may downregulate PCD, as recently autophagy and plant PCD have been shown to be intimately linked.^41, 42^

In summary, our results demonstrate a central role for cathepsin B in plant PCD that might originate in an ancestral PCD pathway pre-existing the divergence of the plant and animal kingdoms. A difference however would be that plant cathepsin B has retained a major role in PCD regulation, whereas in animals the protease may have taken a more restricted role because of the invention/evolution of caspases. As a general point, the involvement of cathepsin B in plant PCD shows that proteases with broad specificity can be part of a PCD cascade. It remains to refine our knowledge of the cathepsin B-mediated PCD process in plants and to position cathepsin B in relation to other cell death proteases.

## Materials and Methods

### Plant material

Seeds of *A. thaliana* ecotype Columbia (Col-0) and transgenic knockout lines *atcathb1* (SALK_49118), *atcathb2* (SALK_89030) and *atcathb3* (SALK_19630) in a Col-0 background were obtained from NASC (Nottingham, UK). The *atcathb* double knockout lines, *atcathb 1 × 3* and *atcathb 2 × 3*, were generated by crossing and lack of expression checked using RT-PCR. Finally, the *atcathb* triple-mutant line 62 has been used and described in McLellan *et al.*^18^.

### Purification and identification of caspase-3-like (DEVDase) activity

The 2-week-old *Arabidopsis* seedlings grown *in vitro* were irradiated with 50 kJ/m^2^ UV-C (CL-1000, UVP, Upland, CA, USA). After 1 h of incubation in continuous light, 80 g of seedlings were ground in liquid nitrogen and total proteins were extracted for 1 h at 4 °C in 0.2 M NaCl, 3 mM DTT, 50 mM CH_3_COONa, pH 5, ratio 1.5 g/5 ml. The extract was filtered through a 0.45 *μ*m filter unit and loaded onto a 60-ml bacitracin-sepharose column (bacitracin from Sigma (St. Louis, MO, USA), activated sepharose from Amersham Biosciences, Amersham, UK). After washing off unbound proteins, the bacitracin-bound DEVDase was eluted by distilled water with 3 mM DTT. The purified DEVDase fraction was incubated with biotin-DEVD–FMK (Bachem Ltd, Bubendorf, Switzerland) at final concentrations of 1 or 5 *μ*M for 1 h at 37 °C in 100 mM NaCl, 25 mM NaOAc, 3 mM DTT, 100 *μ*M PMSF, pH 5.5. Proteins were then precipitated by adding bovine serum albumin (BSA) at 1 mg/ml as a carrier and 10% final trichloroacetic acid (TCA) and kept on ice for 30 min. The precipitated proteins were collected by centrifugation (7800 × *g*, 20 min, 4 °C). The protein pellet was washed once with 70% acetone and the dried pellet resuspended in TBS (20 mM Tris, 137 mM NaCl, pH 7.6). Then, 6.6 *μ*l of streptavidin-polystyrene magnetic beads (M-280, Dynal, Carlsbad, CA, USA) were added per 1 ml resuspended proteins to capture the biotinylated DEVDase and washed seven times: twice with TBS; twice with TBS, 1 M NaCl; twice with TBS, 1% Triton X-100; and once with distilled water. After washing, the beads were resuspended in 1 × alkaline SDS loading buffer (pH 8). SDS-PAGE gels were stained using a MS/MS-compatible silver stain (Pierce, Waltham, MA, USA) and cut-out bands sent for LC-MS/MS analysis.

### Biotin-DEVD pull-down from whole protein extracts

A total protein extract was prepared from 10 g of *Arabidopsis* leaves (~5–6-week-old seedlings in short day) in 10 ml cold extraction buffer (H_2_O, 3 mM DTT, 200 *μ*M PMSF) at 4 °C. After centrifugation at 18 000 × *g*, 4 °C, 15 min, the supernatant was labelled with 25 *μ*M Biotin-DEVD–FMK for 1 h and filtered (0.22 *μ*m). The labelling buffer was replaced with PBS using 5 repeated spins and a 20 ml Pierce Protein Concentrator, 9K MWCO. The sample was incubated with 100 *μ*l magnetic streptavidin beads (M280, Life Technologies, Carlsbad, CA, USA) at RT for 1 h. After six washes, the beads and bound proteins were boiled in 30 *μ*l SDS loading buffer at 95 °C for 5 min before SDS-PAGE and silver staining.

### LC-MS protein identification

Protein bands of interest were excised from gels, reduced with 10 mM dithiothreitol and alkylated with 55 mM iodoacetamide. Samples were digested with trypsin overnight at 37 °C and analysed by LC-MS/MS using a NanoAcquity LC (Waters, Wilmslow, UK) coupled to a 4000 Q-TRAP (Applied Biosystems, Foster City, CA, USA). Peptides were selected for fragmentation automatically by data-dependent analysis. Data produced were searched using Mascot (Matrix Science, London, UK), against the Uniprot database with *Arabidopsis* as selected taxonomy. Mass spectrometry identification in this project was carried out by the Biomolecular Analysis Facility, University of Manchester (Manchester, UK) or the Taplin Mass Spectrometry Facility, Harvard Medical School (Boston, MA, USA). N-terminal sequencing was carried out at the Faculty of Biological Sciences (Leeds, UK).

### Plasmids

The plasmids containing the cDNA of *AtCathB-3* (pR17098) was obtained from RIKEN BRC (Tsukuba, Japan) and ABRC (Columbus, OH, USA). The signal peptide (25 residues) of AtCathB-3 were deleted using PCR and cloned into the *E. coli* expression vector pSCherry2 (Delphi Genetics, Charleroi, Belgium) that added a His-tag at the C-terminus and a cherry-tag at the N-terminus to produce pSCherry::AtCB3. Cherrytag encodes a red polypeptide (heme-binding part of cytochrome, 11 kDa) providing a visual aid for estimating solubility (Delphi). The DNA sequences of Cherry-AtCB3-6 × His were then amplified and ligated into the baculovirus transfer vector pAcGP67A (Pharmingen, Oxford, UK) at the *Xba*I and *Pst*I sites to give pAcGP67A::AtCB3. A null allele of AtCathB-3 with a C_131_A mutation in the active site was generated using the Quikchange (Stratagene, Santa Clara, CA, USA) protocol and the primers 1610QF and 1610QR to give pAcGP67A-AtCB3C_131_A. The ORF of *AtCathB3* without a stop codon was amplified and cloned into pENTR1A (Invitrogen, Carlsbad, CA, USA) to give pENTR1A::AtCB3nostop. pENTR1A::AtCB3nostop was subsequently recombined into pH7RWE (VIB, Ghent, Belgium) to generate a AtCathB3::mRFP fusion construct for plant expression. The sequences of primers used are in Supplementary Table S2.

### Recombinant expression in insect cells of AtCathB

Baculovirus secretory transfer vectors pAcGP67A-AtCB3 and pAcGP67A-AtCB3C_131_A containing Cherrytag::CathepsinB::His fusions were transfected along with linearised baculovirus DNA (OET-cathepsin deleted strain) into Sf9 insect cells grown in Excell-420 serum-free medium (Sigma) using Genejuice liposome transfection reagent  (Novagen, Darmstadt, Germany). Next, 50 ml of Hi-5 cells at 2.5 × 10^6^ cells per ml were infected with CathepsinB P2 virus high titre stock. The media containing secreted AtCathB were harvested at 72 h post infection, before cell lysis, by removing the cells at 1000 × *g* for 10 min at 4 °C. Imidazole was added to the media (5 mM final) and purified on Ni-NTA resin (Qiagen, Hilden, Germany), with slow mixing of the media/bead suspension at 4 ^o^C for 2 h. After washes using a column and imidazole gradient between 5 and 15 mM (W1 to W5) in 25 mM Tris-HCl, pH 8, and 150 mM NaCl, recombinant AtCathB was eluted with 100 mM imidazole buffer and fractions collected. All fractions were analysed by SDS-PAGE. After elution, purified AtCathB was stored at −20 °C until activation.

### Self-activation of recombinant AtCathB

Unless stated otherwise, aliquots of purified recombinant AtCathB at 1 *μ*g/μl were transferred in 2 ml centrifuge tubes. The pH of the sample was brought down to pH 4.5 by adding the same volume of McIlvaine buffer pH 4.5 (10.65 ml of 0.1 M citric acid and 9.35 ml of 0.2 M disodium hydrogenphosphate in 20 ml final volume) to the sample. The recombinant protein was then left at 4 °C for self-activation for 3–7 days in the absence or presence of 10 *μ*g/ml dextran sulphate as previously described.^32^ Activation and sizes were verified using SDS-PAGE.

### Enzymatic activity assay

Enzymatic activities were carried out in assay buffer 100 mM NaCl, 25 mM NaOAc, 3 mM DTT and 100 *μ*M PMSF in whole extract only, pH 5.5, using synthetic fluorogenic substrates at 50 to 100 *μ*M and a Fluoroskan Ascent microplate fluorometer (Labsystems, DYNEX Technologies, Chantilly, VA, USA). Ex 355 nm and Em 460 nm were used for substrates conjugated with 7-amino-4-methylcoumarin (AMC). Ex 485 nm and Em 538 nm were used for ac-DEVD_2_-rhodamine 110 (Bachem). Slopes were calculated out of 15 measures every 2 min and expressed as fluorescent unit/min per mg protein. Inhibitors were incubated at various final concentrations for 30 min before the enzymatic activity assay.

### Western blot and activity labelling

Proteins were separated on SDS-PAGE (15%) and transferred to a Hybond-P membrane (Amersham Biosciences) at 100 V for 1 h in transfer buffer. The membrane was then blocked with BSA at 4 °C overnight. After incubation with anti-His (Amersham) followed by secondary antibody incubation, the membrane was treated with a mixture of supersignal (Thermo, Waltham, MA, USA) west-femto substrate and west-pico substrate (1 : 5 vol/vol). Pierce imaging film was used and developed with a Compact 2 processor. Activity probe labelling was carried out in 100 mM NaCl, 25 mM NaOAc, 3 mM DTT, pH 5.5, using biotin-DEVD–FMK at 2–50 *μ*M final concentration, at 37 °C for 60 min. Protein were separated on SDS-PAGE (15%) and transferred to a Hybond-P membrane (Amersham Biosciences). After incubation with streptavidin-horseradish peroxidase (HRP) (Amersham), the biotinylated proteins were detected using a mixture of supersignal (Thermo) west-femto substrate and west-pico substrate (1 : 5 vol/vol). Apparent size was calculated using GelAnalyser (www.gelanalyser.com); kDa brackets are given when apparent sizes varied slightly between gel repeats, as expected.

### Protoplast isolation and PCD detection

Protoplasts were prepared essentially as in Danon *et al.*^14^ PCD was induced by 10 kJ/m^2^ UV-C, 10 mM H_2_O_2_ or 15 *μ*g/ml tunicamycin. PCD was detected using Evans blue (0.05%) or Sytox green (5 *μ*M). The percentage of dye-positive protoplasts over the total protoplasts was calculated from triplicate.

### UV-C treatment of seedlings

This death assay was carried out essentially as in Danon *et al.*^14^ Sterile seeds were plated *in vitro* on solid MS media without glucose and germinated in 16 h light at 22 °C. At 4 to 7 days, seedlings were treated with 10 kJ/m^2^ UV-C using a CL-1000 UV crosslinker (UVP). Bleaching was scored at 1 day.

### Methyl viologen treatment of seedlings

Sterile seeds were plated *in vitro* on solid MS media without glucose, supplemented with methyl viologen (Sigma) at various concentrations from 0.25 to 10 *μ*M and germinated in continuous light at 22 °C. The percentage of seedlings with green cotyledons over total seed was scored at 10 days. For mutant lines, 5 *μ*M was found to give the clearest phenotype.

## Figures and Tables

**Table 1:** Effect of inhibitors on the DEVDase activity of recombinant AtCathB3

**Inhibitor**	**Concentration**	**Remaining activity (%)**
Control	N/A	100±4.3
FR-FMK	100 *μ*M	0
LVK-CHO	100 *μ*M	0
CA-074	1 mM	0
DEVD-CHO	100 **μ**M	0.3±0.0
YVAD-CHO	100 *μ*M	43.3±0.6

## Abbreviations

AtCathB: *Arabidopsis thaliana* cathepsin B
CHO: aldehyde (pseudo acid)
DCG-04: cysteine protease probe
DEVD: Asp-Glu-Val-Asp
ER: endoplasmic reticulum
FA: Phe-Ala
FMK: fluoromethylketone
HR: hypersensitive response
HRP: horseradish peroxidase
IETD: Ile-Glu-Thr-Asp
LC-MS/MS: liquid chromatography with tandem mass spectrometry
LVK: Leu-Val-Lys
MV: methyl viologen
MW: molecular weight
PBA1: *β*1 subunit of the 20S proteasome
PCD: programmed cell death
ROS: reactive oxygen species
RR: Arg-Arg
UV-C: ultraviolet-C
VEID: Val-Glu-Ile-Asp
WT: wild type
YVAD: Tyr-Val-Ala-Asp

## Supplementary Material

Supplementary Informations

## References

[bib1] Drury GE, Gallois PProgrammed cell death in plants and flowers. In: da Silva Teixeira AJ (ed) Floriculture, Ornamental and Plant Biotechnology. Global Science Books, Ltd, 2006 pp 141–156.

[bib2] van Doorn WG, Beers EP, Dangl JL, Franklin-Tong VE, Gallois P, Hara-Nishimura I et al. Morphological classification of plant cell deaths. Cell Death Differ 2011; 18: 1241–1246. 21494263 10.1038/cdd.2011.36 PMC3172093

[bib3] Rotari VI, He R, Gallois P. Death by proteases in plants: whodunit. Physiol Plantarum 2005; 123: 376–385.

[bib4] Kacprzyk J, Daly CT, McCabe PF. The botanical dance of death: programmed cell death in plants. Adv Bot Res 2011; 60: 169–261.

[bib5] del Pozo O, Lam E. Caspases and programmed cell death in the hypersensitive response of plants to pathogens. Curr Biol 1998; 8: 1129–1132. 9778530 10.1016/s0960-9822(98)70469-5

[bib6] Bonneau L, Ge Y, Drury GE, Gallois P. What happened to plant caspases? J Exp Bot 2008; 59: 491–499. 18272922 10.1093/jxb/erm352

[bib7] Tsiatsiani L, Van Breusegem F, Gallois P, Zavialov A, Lam E, Bozhkov PV. Metacaspases. Cell Death Differ 2011; 18: 1279–1288. 21597462 10.1038/cdd.2011.66 PMC3172103

[bib8] Hatsugai N, Iwasaki S, Tamura K, Kondo M, Fuji K, Ogasawara K et al. A novel membrane fusion-mediated plant immunity against bacterial pathogens. Genes Dev 2009; 23: 2496–2506. 19833761 10.1101/gad.1825209 PMC2779742

[bib9] Gu C, Kolodziejek I, Misas-Villamil J, Shindo T, Colby T, Verdoes M et al. Proteasome activity profiling: a simple, robust and versatile method revealing subunit-selective inhibitors and cytoplasmic, defense-induced proteasome activities. Plant J 2009; 62: 160–170. 20042019 10.1111/j.1365-313X.2009.04122.x

[bib10] Han J-J, Lin W, Oda Y, Cui K-M, Fukuda H, He X-Q. The proteasome is responsible for caspase-3-like activity during xylem development. Plant J 2012; 72: 129–141. 22680239 10.1111/j.1365-313X.2012.05070.x

[bib11] Vacca RA, Valenti D, Bobba A, Merafina RS, Passarella S, Marra E. Cytochrome c is released in a reactive oxygen species-dependent manner and is degraded via caspase-like proteases in tobacco Bright-Yellow 2 cells en route to heat shock-induced cell death. Plant Physiol 2006; 141: 208–219. 16531480 10.1104/pp.106.078683 PMC1459318

[bib12] Kim M, Ahn JW, Jin UH, Choi D, Paek KH, Pai HS. Activation of the programmed cell death pathway by inhibition of proteasome function in plants. J Biol Chem 2003; 278: 19406–19415. 12637532 10.1074/jbc.M210539200

[bib13] Rozman-Pungercar J, Kopitar-Jerala N, Bogyo M, Turk D, Vasiljeva O, Stefe I et al. Inhibition of papain-like cysteine proteases and legumain by caspase-specific inhibitors: when reaction mechanism is more important than specificity. Cell Death Differ 2003; 10: 881–888. 12867995 10.1038/sj.cdd.4401247

[bib14] Danon A, Rotari VI, Gordon A, Mailhac N, Gallois P. Ultraviolet-C overexposure induces programmed cell death in Arabidopsis, which is mediated by caspase-like activities and which can be suppressed by caspase inhibitors, p35 and Defender against Apoptotic Death. J Biol Chem 2004; 279: 779–787. 14573611 10.1074/jbc.M304468200

[bib15] Rotari VI, Dando PM, Barrett AJ. Legumain forms from plants and animals differ in their specificity. Biol Chem 2001; 382: 953–959. 11501761 10.1515/BC.2001.119

[bib16] Zakharov A, Carchilan M, Stepurina T, Rotari VI, Wilson K, Vaintraub I. A comparative study of the role of the major proteinases of germinated common bean (Phaseolus vulgaris L.) and soybean (Glycine max (L.) Merrill) seeds in the degradation of their storage proteins. J Exp Bot 2004; 55: 2241–2249. 15333645 10.1093/jxb/erh247

[bib17] Rozman-Pungercar J, Caglic D, Sajid M, Dolinar M, Vasiljeva O, Požgan U et al. Autocatalytic processing of procathepsin B is triggered by proenzyme activity. FEBS J 2008; 276: 660–668. 10.1111/j.1742-4658.2008.06815.x PMC4551429 19143833

[bib18] McLellan H, Gilroy EM, Yun B-W, Birch PRJ, Loake GJ. Functional redundancy in the Arabidopsis Cathepsin B gene family contributes to basal defence, the hypersensitive response and senescence. New Phytol 2009; 183: 408–418. 19453434 10.1111/j.1469-8137.2009.02865.x

[bib19] Danon A, Gallois P. UV-C radiation induces apoptotic-like changes in Arabidopsis thaliana. FEBS Lett 1998; 437: 131–136. 9804186 10.1016/s0014-5793(98)01208-3

[bib20] Gao C, Xing D, Li L, Zhang L. Implication of reactive oxygen species and mitochondrial dysfunction in the early stages of plant programmed cell death induced by ultraviolet-C overexposure. Planta 2008; 227: 755–767. 17972096 10.1007/s00425-007-0654-4

[bib21] Chen S, Dickman MB. Bcl-2 family members localize to tobacco chloroplasts and inhibit programmed cell death induced by chloroplast-targeted herbicides. J Exp Bot 2004; 55: 2617–2623. 15475374 10.1093/jxb/erh275

[bib22] Chichkova NV, Shaw J, Galiullina RA, Drury GE, Tuzhikov AI, Kim SH et al. Phytaspase, a relocalisable cell death promoting plant protease with caspase specificity. EMBO J 2010; 29: 1149–1161. 20111004 10.1038/emboj.2010.1 PMC2845272

[bib23] He R, Drury GE, Rotari VI, Gordon A, Willer M, Farzaneh T et al. Metacaspase-8 modulates programmed cell death induced by ultraviolet light and H2O2 in Arabidopsis. J Biol Chem 2008; 283: 774–783. 17998208 10.1074/jbc.M704185200

[bib24] Darehshouri A, Affenzeller M, Meindl UL. Cell death upon H2O2 induction in the unicellular green alga Micrasterias. Plant Biol 2008; 10: 732–745. 18950431 10.1111/j.1438-8677.2008.00078.x PMC2923030

[bib25] Jiao J, Sun L, Zhou B, Gao Z, Hao Y, Zhu X et al. Hydrogen peroxide production and mitochondrial dysfunction contribute to the fusaric acid-induced programmed cell death in tobacco cells. J Plant Physiol 2014; 171: 1197–1203. 24973592 10.1016/j.jplph.2014.03.015

[bib26] Tiwari BS, Belenghi B, Levine A. Oxidative stress increased respiration and generation of reactive oxygen species, resulting in ATP depletion, opening of mitochondrial permeability transition, and programmed cell death. Plant Physiol 2002; 128: 1271–1281. 11950976 10.1104/pp.010999 PMC154255

[bib27] Belenghi B, Acconcia F, Trovato M, Perazzolli M, Bocedi A, Polticelli F et al. AtCYS1, a cystatin from Arabidopsis thaliana, suppresses hypersensitive cell death. Eur J Biochem 2003; 270: 2593–2604. 12787025 10.1046/j.1432-1033.2003.03630.x

[bib28] Zuppini A, Navazio L, Mariani P. Endoplasmic reticulum stress-induced programmed cell death in soybean cells. J Cell Sci 2004; 117: 2591–2598. 15159454 10.1242/jcs.01126

[bib29] Watanabe N, Lam E. BAX inhibitor-1 modulates endoplasmic reticulum stress-mediated programmed cell death in Arabidopsis. J Biol Chem 2008; 283: 3200–3210. 18039663 10.1074/jbc.M706659200

[bib30] Yang Z-T, Wang M-J, Sun L, Lu S-J, Bi D-L, Sun L et al. The membrane-associated transcription factor NAC089 controls ER-stress-induced programmed cell death in plants. PLoS Genet 2014; 10: e1004243. 24675811 10.1371/journal.pgen.1004243 PMC3967986

[bib31] Tsuji A, Kikuchi Y, Ogawa K, Saika H, Yuasa K, Nagahama M. Purification and characterization of cathepsin B-like cysteine protease from cotyledons of daikon radish, Raphanus sativus. FEBS J 2008; 275: 5429–5443. 18959767 10.1111/j.1742-4658.2008.06674.x

[bib32] Niemer M, Mehofer U, Verdianz M, Porodko A, Schähs P, Kracher D et al. Nicotiana benthamiana cathepsin B displays distinct enzymatic features which differ from its human relative and aleurain-like protease. Biochimie 2015; 122: 119–125. 26166069 10.1016/j.biochi.2015.06.017

[bib33] Illy C, Quraishi O, Wang J, Purisima E, Vernet T, Mort JS. Role of the occluding loop in cathepsin B activity. J Biol Chem 1997; 272: 1197–1202. 8995421 10.1074/jbc.272.2.1197

[bib34] Iglesias-Fernández R, Wozny D, Iriondo-de Hond M, Oñate-Sánchez L, Carbonero P, Barrero-Sicilia C. The AtCathB3 gene, encoding a cathepsin B-like protease, is expressed during germination of Arabidopsis thaliana and transcriptionally repressed by the basic leucine zipper protein GBF1. J Exp Bot 2014; 65: 2009–2021. 24600022 10.1093/jxb/eru055 PMC3991739

[bib35] Canbay A, Guicciardi ME, Higuchi H, Feldstein A, Bronk SF, Rydzewski R et al. Cathepsin B inactivation attenuates hepatic injury and fibrosis during cholestasis. J Clin Invest 2003; 112: 152–159. 12865404 10.1172/JCI17740 PMC164289

[bib36] Guicciardi ME, Miyoshi H, Bronk SF, Gores GJ. Cathepsin B knockout mice are resistant to tumor necrosis factor-α-mediated hepatocyte apoptosis and liver injury. Am J Pathol 2001; 159: 2045–2054. 11733355 10.1016/s0002-9440(10)63056-8 PMC1850591

[bib37] Cirman T, Oresić K, Mazovec GD, Turk V, Reed JC, Myers RM et al. Selective disruption of lysosomes in HeLa cells triggers apoptosis mediated by cleavage of Bid by multiple papain-like lysosomal cathepsins. J Biol Chem 2004; 279: 3578–3587. 14581476 10.1074/jbc.M308347200

[bib38] Repnik U, Stoka V, Turk V, Turk B. Lysosomes and lysosomal cathepsins in cell death. Biochim Biophys Acta 2012; 1824: 22–33. 21914490 10.1016/j.bbapap.2011.08.016

[bib39] Boya P, Kroemer G. Lysosomal membrane permeabilization in cell death. Oncogene 2008; 27: 6434–6451. 18955971 10.1038/onc.2008.310

[bib40] Carter C, Pan S, Zouhar J, Avila EL, Girke T, Raikhel NV. The vegetative vacuole proteome of Arabidopsis thaliana reveals predicted and unexpected proteins. Plant Cell 2004; 16: 3285–3303. 15539469 10.1105/tpc.104.027078 PMC535874

[bib41] Minina EA, Bozhkov PV, Hofius D. Autophagy as initiator or executioner of cell death. Trends Plant Sci 2014; 19: 692–697. 25156061 10.1016/j.tplants.2014.07.007

[bib42] Coll NS, Smidler A, Puigvert M, Popa C, Valls M, Dangl JL. The plant metacaspase AtMC1 in pathogen-triggered programmed cell death and aging: functional linkage with autophagy. Cell Death Differ 2014; 21: 1399–1408. 24786830 10.1038/cdd.2014.50 PMC4131171

