# Supplementary material for: Inhibition of cathepsin B by caspase-3 inhibitors blocks programmed cell death in Arabidopsis
Source: Cell Death Differ. 2016 Apr 8;23(9):1493–501. doi: 10.1038/cdd.2016.34 (PMC5072426; doi:10.1038/cdd.2016.34)
Supplement: Supplementary Informations [file cdd201634x1.pdf]

Supplementary figures:

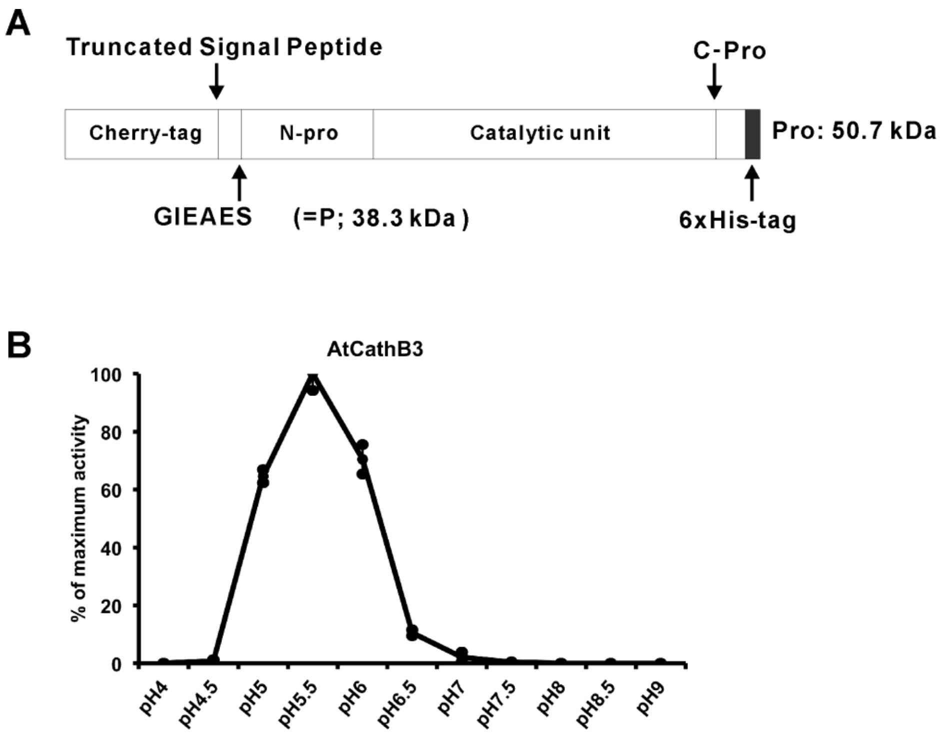

Figure S1. Recombinant AtCathB-3. A) Cartoon of the AtCathB3 construct expressed in insect cells. GIEAES: N terminal sequence of the P form in Fig 2C, predicted MW indicated. B) Optimal pH of AtCathB3 is 5.5. Activated recombinant AtCathB3 was assayed for DEVDase activity at a range of pH values. Results are given as percentage of maximum activity measured.

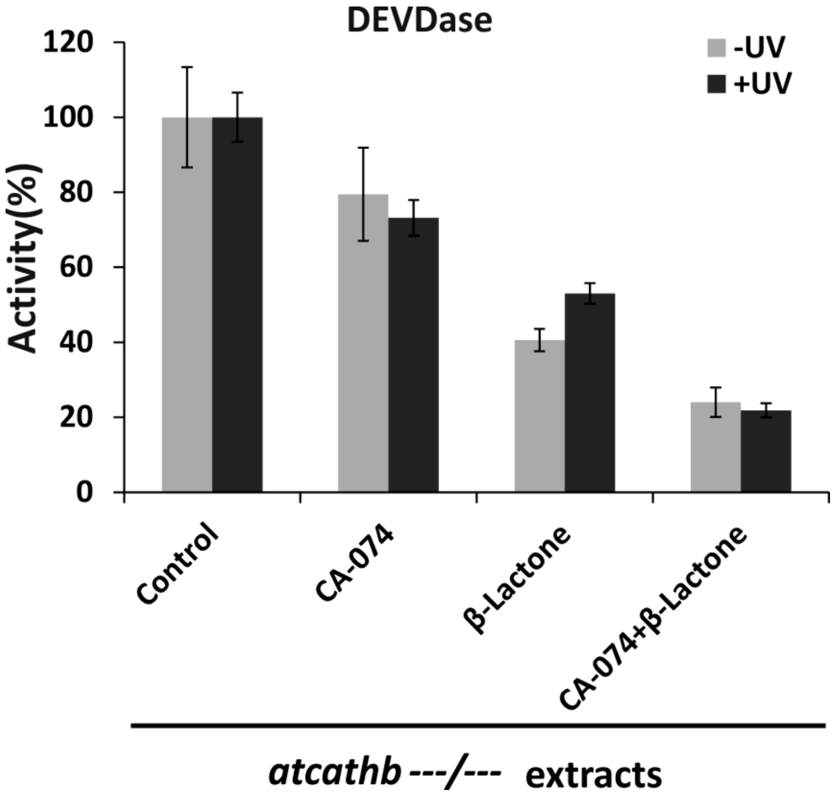

Figure S2. Contribution of the proteasome to the casp3-like activity in a cathepsin B triple mutant. Seedlings of the triple mutant line (atcathb---/---) were not treated (- UV) or treated with 10 kJ/m^2 UV-C (+ UV) to induce PCD. After 24h, total protein extracts were pre-incubated with various protease inhibitors at the final concentration of 100 μM (except CA-074 concentration was 1 mM) for 30 min at 30 °C, before measuring the caspase-3-like activity over 10-15 time points using DEVD as a substrate. The remaining activity is calculated as a percentage of the relevant no inhibitor control. CA-074: cathepsin B inhibitor, ß-lactone: proteasome inhibitor. Error bars indicate ±S.D. value for triplicates.

A

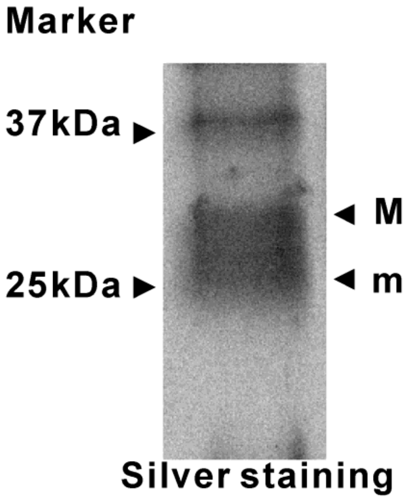

B

Band	Peptide hits		
	CathB1	CathB2	CathB3
M	0	4	7
m	0	1	3

Figure S3. Protein identification in biotin-DEVD-FMK pull-down from soluble protein extract. A) A soluble protein extract from 10g of Col-0 leaves was labelled using biotin-DEVD-FMK, pulled down using streptavidin beads and separated by SDS-PAGE. Protein were visualised by silver staining. The positions of two protein markers are indicated (kDa). B) The two visible bands at 33kDa and 25 kDa (M and m) were excised and analysed using LC/MS/MS. The numbers of peptide hits for the three AtCathB paralogues present/absent in the protein list for each bands are indicated suggesting the 25kDa band is a further processed form of the 33 kDa band.

A 33 kDa band

At4g01610
MAVYNTKLCL ASVFLLLGLL LAFDLKGIEA ESLTKQKLDS KILQDEIVKK
VNENPNAGWK AAINDRFSNA TVAEFKRLLG VKPTPKKHFL GVPIVSHDPS
LKLPKAFDAR TAWPQCTSIG NILDQGHCGS CWAFGAVESL SDRFCIQFGM
NISLSVNDLL ACCGFRCGDG CDGGYPIAAW QYFSYSGVVT EECDPYFDNT
GCSHPGCEPA YPTPKCSRKC VSDNKLWSES KHYSVSTYTV KSNPQDIMAE
VYKNGPVEVS FTVYEDFAHY KSGVYKHITG SNIGGHAVKL IGWGTSSEGE
DYWLMANQWN RGWGDDGYFM IRRGTNECGI EDEPVAGLPS SKNVFRVDTG
SNDLPVASV

B 25 kDa band

AT4G01610
MAVYNTKLCL ASVFLLLGLL LAFDLKGIEA ESLTKQKLDS KILQDEIVKK
VNENPNAGWK AAINDRFSNA TVAEFKRLLG VKPTPKKHFL GVPIVSHDPS
LKLPKAFDAR TAWPQCTSIG NILDQGHCGS CWAFGAVESL SDRFCIQFGM
NISLSVNDLL ACCGFRCGDG CDGGYPIAAW QYFSYSGVVT EECDPYFDNT
GCSHPGCEPA YPTPKCSRKC VSDNKLWSES KHYSVSTYTV KSNPQDIMAE
VYKNGPVEVS FTVYEDFAHY KSGVYKHITG SNIGGHAVKL IGWGTSSEGE
DYWLMANQWN RGWGDDGYFM IRRGTNECGI EDEPVAGLPS SKNVFRVDTG
SNDLPVASV

Figure S4. Position of the AtCathB3 peptides identified by LC/MS/MS in biotin-DEVD-fmk pull down from soluble protein extract. The pull-down sample was separated on a SDS-PAGE and silver stained (Fig. S3). Two visible bands were excised at 33kDa and 25 kDa. AtCathB3 was present in both protein list and the position of the peptide hits obtained is represented in red, underlined, for each band. The peptides in the 33kDa band cover the exact length of a predicted mature CathB form (M), from the N-terminal L to the C-terminal K.

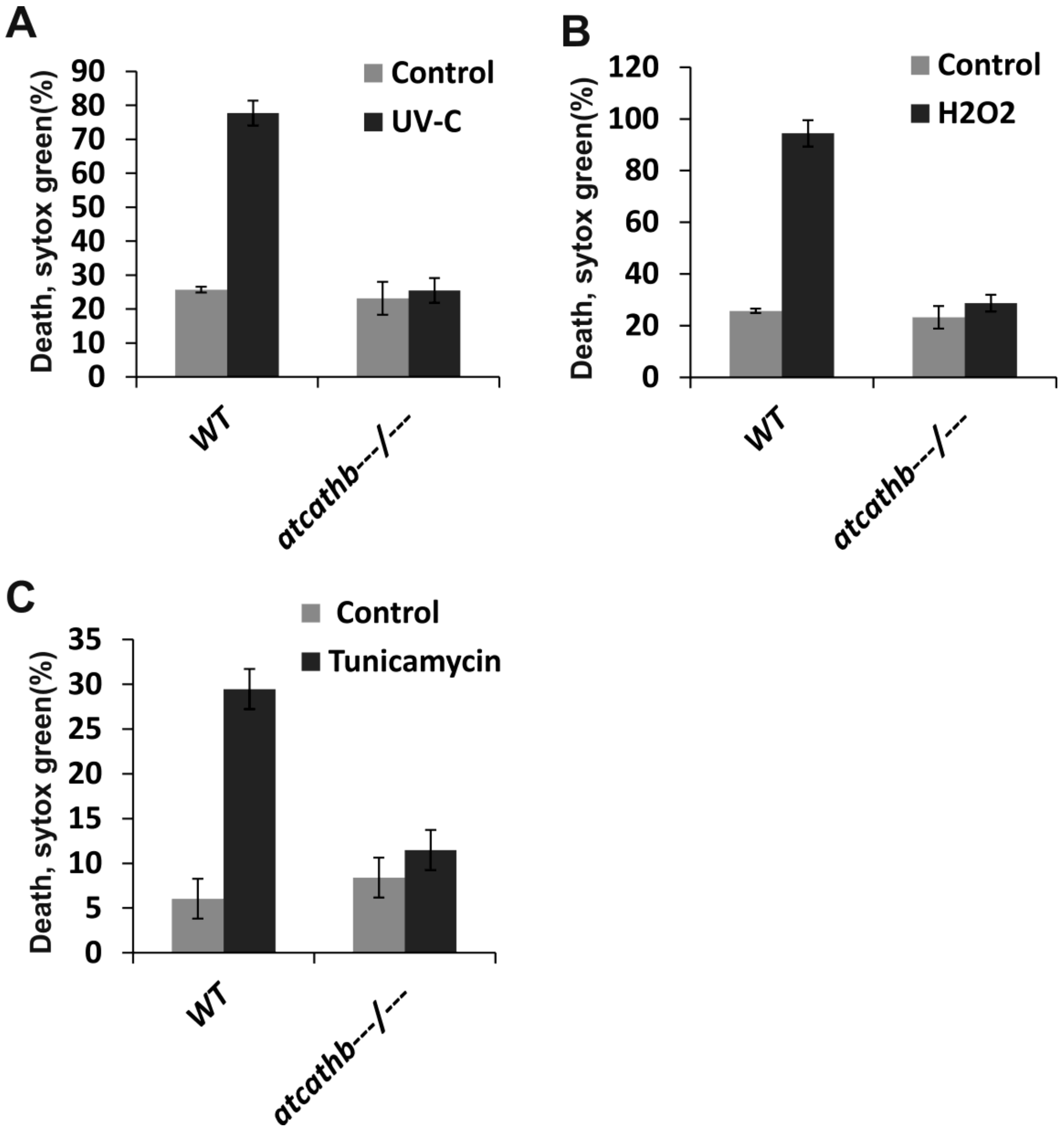

Figure S5. ***AtCathB*** **mediates the PCD phenotype induced in protoplasts by various abiotic stresses.** ***(A) – (C)*** *Arabidopsis* seedlings from WT and cathepsin B triple mutant line (atcathb ---/---) were grown *in vivo* for two weeks. Protoplasts isolated from seedlings were exposed to 10 kJ/m^2 UV-C **(A)**, 10mM H_2O_2 **(B)** or 15µg/mL tunicamycin **(C)** to induce PCD. Protoplasts were subsequently stained with SYTOX green after 4 h continuous light incubation. Results are given as the percentage of SYTOX positive in the population. Error bars indicate ±S.D. for triplicates.

Supplementary tables:

Table S1. Partial purification of caspase-3 like activity from *Arabidopsis* seedlings.

Step	Volume, ml	Protein			Casp-3 like Activity			Specific activity, U/mg	Purification fold
		μg/ml	mg/V	%	U/98 μl	U/V	%		
Extract	120	687.5	82.5	100	0.46	563.3	100	6.8	1
Bacitracin-Sepharose	50	34.7	1.74	2.1	1.47	750	133	431	63

Note: Typical bacitracin purification experiment. 35 g of UV-C treated *Arabidopsis* seedlings homogenate were extracted with 115 ml (1.5 g : 5 ml) 50 mM acetate buffer, pH 5, 0.2M NaCl, 3 mM DTT and the extract loaded on a bacitracin-sepharose column for affinity purification of cysteine proteases.

Table S2. Primers used in this study

Primer name	Sequence
CB3CherryF	GCTCTAGAAATGGCAGAACAAAGCGACAAG
CB3CherryR	AACTGCAGTCAGTGGTGGTGGTGGTGG
CB3F	GGAATTCCCATGGCTGTTTACAATACCAAAC
CB3nostopR	GGGTCTCGAGGTAACCGATGCAACCGGAAGATC
mRFP-FW	GATCCACCGGTCGCCACCATGGCCTCCTCCGAGGACG
mRFP-RW	CCGGAATTCGGCGCCGGTGGAGTGGCGGC
1610QF quikchange	GGA CAT TGT GGT TCT **GCC** TGG GCA TTT GG
1610QR quikchange	CC AAA TGC CCA GGC AGA ACC ACA ATG TCC
